# Repeatome Analysis and Satellite DNA Chromosome Patterns in *Hedysarum* Species

**DOI:** 10.3390/ijms252212340

**Published:** 2024-11-17

**Authors:** Olga Yu. Yurkevich, Tatiana E. Samatadze, Svyatoslav A. Zoshchuk, Alexey R. Semenov, Alexander I. Morozov, Inessa Yu. Selyutina, Alexandra V. Amosova, Olga V. Muravenko

**Affiliations:** 1 Engelhardt Institute of Molecular Biology, Russian Academy of Sciences, 32 Vavilov St., 119991 Moscow, Russia; 2 All-Russian Institute of Medicinal and Aromatic Plants, Federal Agency for Scientific Organizations, 7 Green St., 117216 Moscow, Russia; 3 Central Siberian Botanical Garden, SB RAS, 101 Zolotodolinskaya St., 630090 Novosibirsk, Russia

**Keywords:** next-generation sequencing (NGS), *Hedysarum* L., repeatome, satDNAs, 35S rDNA, 5S rDNA, FISH analysis, chromosome variability

## Abstract

The cosmopolitan genus *Hedysarum* L. (Fabaceae) is divided into sections *Hedysarum*, *Stracheya*, and *Multicaulia.* This genus includes many valuable medicinal, melliferous, and forage species. The species taxonomy and genome relationships within the sections are still unclear. We examined intra- and interspecific diversity in the section (sect.) *Hedysarum* based on repeatome analyses using NGS data, bioinformatic technologies, and chromosome FISH mapping of 35S rDNA, 5S rDNA, and the identified satellite DNA families (satDNAs). A comparison of repeatomes of *H. alpinum*, *H. theinum*, and *H. flavescens* revealed differences in their composition. However, similarity in sequences of most satDNAs indicated a close relationship between genomes within sect. *Hedysarum*. New effective satDNA chromosomal markers were detected, which is important for karyotype analyses within *Hedysarum*. Intra- and interspecific variability in the chromosomal distribution patterns of the studied markers were revealed, and species karyograms were constructed. These results provided new insight into the karyotype structures and genomic diversity within sect. *Hedysarum*, clarified the systematic position of *H. sachalinense* and *H. arcticum*, and confirmed the distant genomic relationships between species from sections *Hedysarum* and *Multicaulia.* Our findings are important for further comparative genome studies within the genus *Hedysarum*.

## 1. Introduction

*Hedysarum* L. is one of the most systematically complicated genera within the legume family (Fabaceae). According to various classifications, this genus includes about 160–200 species [1,2,3]. During the evolutionary process, the *Hedysarum* species have adapted to various ecological niches, including mountainous regions, steppes, and deserts. Many *Hedysarum* species are widely used in traditional medicine. The medicinal plants from the section (sect.) *Hedysarum* and sect. *Multicaulia* contain many antiviral and antibacterial substances, e.g., xanthone mangiferin [4,5,6], which have immunomodulatory, antioxidant, antitumor, and antidiabetic effects [7,8,9,10].

Currently, molecular genetic studies have significantly clarified the phylogenetic relationships within the genus *Hedysarum*. Phylogenetic analysis based on nuclear and plastid DNA sequences identified three well-supported clades in this genus, which were redefined as sections *Hedysarum*, *Stracheya*, and *Multicaulia* [11,12,13,14]. The phylogenetic studies conducted using nrDNA ITS and cpDNA sequences presented two main lineages in the genus *Hedysarum*, the *Hedysarum* s.s. clade (*Hedysarum* sect. *Hedysarum* and *Hedysarum* sect. *Stracheya*) and the *Sartoria* clade (*Hedysarum* sect. *Multicaulia*) [13]. At the same time, Duan et al. [12] and Nafisi et al. [14] placed the *Hedysarum* sect. *Stracheya* together with the *Hedysarum* sect. *Multicaulia* based on the analysis of nuclear markers. These results are consistent with the fact that the species from sect. *Hedysarum* are known to have a basic chromosome number x = 7, and the species from sections *Stracheya* and *Multicaulia* have x = 8 [11,15,16]. Most species from the genus *Hedysarum* are diploid, but polyploid species were also found [15,16,17], and polyploidy was identified in different species populations with a wide geographical distribution. In particular, three levels of ploidy were found in various *H. gmelinii* specimens (2n = 2x = 16, 2n = 4x = 32, and 2n = 6x = 48) using a molecular cytogenetic approach [18].

There is still some disagreement regarding the boundaries of the genus and the status of some species, which requires further research. For example, *H. arcticum* B. Fedtsch. is described either as an independent plant species [1,19] or as a subspecies of *H. hedysaroides* (L.) Schinz et Thell. [2,20]. Many *Hedysarum* species demonstrate high intraspecific genetic diversity, which is especially typical for widespread taxa with broken ranges. RAPD and ISSR analyses proved to be effective methods for clarifying the genetic relationships among and within different populations of *H. sangilense* Krasnoborov et Timokhina distributed in the isolated regions of the Republic of Tuva and Northern Mongolia [21]. Some of the *Hedysarum* species are endemic to certain regions, which should be considered in taxonomic studies as well as for these species’ protection. According to ISSR data, the spatial isolation of some Eastern European populations of *H. grandiflorum*, which was caused by outcrops of chalk and limestone, led to local genetic differentiation [22].

Satellite DNA families (satDNAs) were shown to cluster in certain heterochromatin-rich chromosomal regions. In this regard, different tandem DNA repeats are currently used to identify chromosomes in plant karyotypes and clarify species taxonomy [23,24,25]. Significant copy number variability and variations in satDNA sequences were detected even in closely related species, which correlated with genome plasticity and the presence of chromosomal rearrangements [26,27]. Knowledge of the organization of tandem DNA repeats in repeatomes of related species helps identify changes occurring in their genomes during speciation and specify the systematic position of different plant taxa [27,28,29]. In our previous study, we identified various satellite DNA families in repeatomes of several species from sect. *Multicaulia* of the genus *Hedysarum*. FISH mapping of these satDNAs on metaphase chromosomes of species from the sect. *Multicaulia* allowed us to identify the chromosomes in karyotypes and reveal interspecific differences in their genomes [18]. However, the relationships between the genomes of the species from sect. *Multicaulia* and sect. *Hedysarum*, having different chromosome numbers, remain unclear.

In this study, we examined the repeatome compositions of three *Hedysarum* species, *H. alpinum*, *H. flavescens*, and *H. theinum*, using the data of low-coverage genome sequencing (BGI technology) and the RepeatExplorer2/TAREAN pipeline. Based on chromosome FISH mapping of the identified satDNAs as well as classical markers, 35S rDNA and 5S rDNA, we analyzed intra- and interspecific diversity in eight *Hedysarum* species from sect. *Hedysarum*. The results of the present study could provide new insight into the karyotype structures of the species from sect. *Hedysarum*, which is important for further comparative studies of genomes of the species from both sections *Multicaulia* and *Hedysarum*.

## 2. Results

### 2.1. Geographical Distribution of the Studied Hedysarum Species

**Figure 1:**
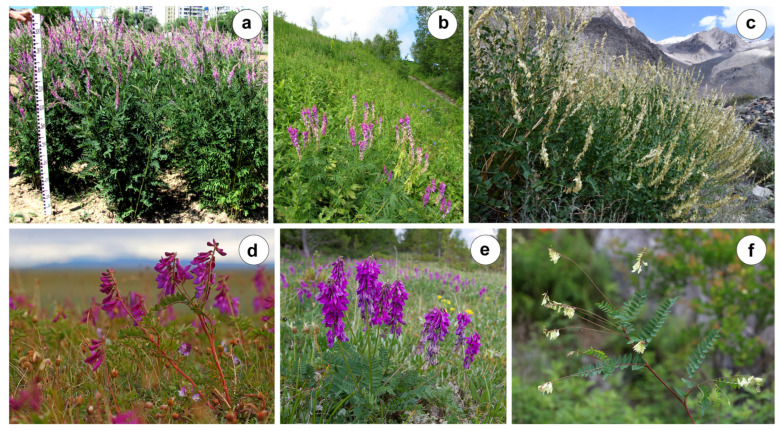
*Hedysarum alpinum* plants growing on the trial plot of AIMAP (Moscow, Russia) (**a**). Wild populations of *H. theinum* (Ivanovsky ridge, Kazakhstan) (**b**), *H. flavescens* (the Tanymas River valley, Tajikistan) (**c**), *H. arcticum* (tundra, the Lena River delta, Republic of Sakha (Yakutia), Russia) (**d**), *H. consaguineum* (Altai Republic, Russia) (**e**), and *H. ussuriense* (Primorsky Krai, Russia) (**f**). The images were taken by S. Romashkina (**a**), I. Selyutina (**b**), V. Pankratov (**c**), D. Kochetkov (**d**), S. Cherenkov (**e**), and V. Volkotrub (**f**).

The studied species have different ranges of their growing areas and different ecological and cenotic features. *Hedysarum alpinum* L. is a species with a wide range that extends from Eastern Europe to North Korea and the Indian subcontinent and from subarctic America to the North American states [1,2,3]. This species grows in wet meadows and in the areas of sparse forests and bushes along river valleys [19]. The range of *H. hedysaroides* (L.) Schinz et Thell. extends from subarctic and subalpine Eurasia to Alaska [2,20]. The type subspecies is distributed in the alpine belt of the Pyrenees, Alps, Sudetes, and Carpathians. *H. arcticum* B. Fedtsch grows in the Arctic and adjacent forest zones, mountain tundras of Arctic Europe, and the Arctic part of Eastern Siberia [1,19]. *H. consaguineum* DC. and *H. theinum* Krasnob. grow in the high mountain zone in the south of Western and Central Siberia and in Eastern Kazakhstan; the range of *H. theinum* extends into Western Mongolia and Central Asia [19,30]. The typical habitats of *H. consaguineum* are alpine and subalpine meadows, rocky placers, and tundras. *H. theinum* is distributed in the alpine and subalpine meadows and adjacent areas of the forest belt. The ecological niches of these two species may sometimes overlap. *H. flavescens* Regel et Schmalh. is widespread in Central Asia (Western Tian Shan, Pamir-Alay Mountains), the Hindu Kush, and Xinjiang, where it grows in the subalpine meadows at an altitude of 2500–3100 m [3,31]. *H. sachalinense* B. Fedtch. is widespread on Sakhalin Island, where it grows on the coastal rocky slopes, occasionally entering the goletsky belt [32]. *H. ussuriense* Schischk. et Kom. is endemic to the Southern Sikhote-Alin, found in the south of Primorsky Krai, where it is found in several localities within the carbonate rocks [32]. At the same time, Chang et al. reported that the range of this species extends from the Russian Far East to Central China and North Korea and to Northern and Central Japan [33]. In Figure 1, several studied species of the *Hedysarum* sect. *Hedysarum* growing in their natural habitat and on the trial plot of AIMAP (All-Russian Institute of Medicinal and Aromatic Plants) are presented.

### 2.2. Satellite Repeat Identification by RepeatExplorer/TAREAN Pipelines

**Figure 2:**
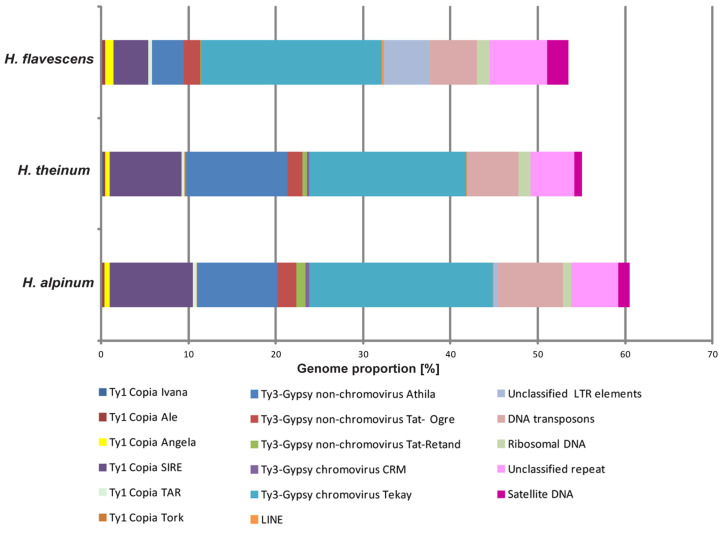
Genome proportions of the most abundant DNA repeats in *H. alpinum*, *H. theinum*, and *H. flavescens*. The genome proportion of the individual repeat type was obtained as a ratio of reads specific to individual repeat types to all reads used for clustering analyses by the RepeatExplorer pipelines.

For the comparative study of repeatomes of diploid species *H. alpinum*, *H. flavescens*, and *H. theinum*, a comprehensive bioinformatic analysis of the genomic read arrays, obtained by high-throughput sequencing based on BGI technology, was carried out by RepeatExplorer/TAREAN pipelines [34]. It was found that transposable elements make up the largest proportion of repetitive DNA. Depending on the species, 38.05–45.44% of them belonged to the mobile elements of Class 1, and 5.45–7.44% of them were mobile elements of Class 2 (with EnSpm CACTA predominating) (Figure 2).

LTR retrotransposons were the most abundant mobile elements of Class 1. Among them, the Ty3-gypsy superfamily was 26.84–33.84% (mostly non-chromovirus Athila and chromovirus Tekay), and the Ty1-copia superfamily was 5.8–10.99% (mostly SIRE) (Supplementary Table S1). The largest number of these elements was revealed in *H. alpinum*. In the genome of *H. theinum*, the highest content of ribosomal DNA (1.45%) was found. Satellite repeats constituted a small fraction of these species’ genomes (0.89–2.41%), and *H. flavescens* had the maximum amount of these repeats. Using TAREAN (Tandem Repeat Analyzer), 6–9 high-confidence putative satellites were revealed (the maximum ones (9) were detected in *H. flavescens*) in the genomes of the studied species. Among them, seven promising putative DNA satellites, which could be potential cytogenetic markers for *Hedysarum*, were identified.

The genome proportion of each tandem DNA repeat and other details, including their consensus length, are shown in Table 1. Each tandem repeat was designated according to the species name: HF for *H. flavescens*, HA for *H. alpinum*, HT for *H. theinum*, and the numerical name of a particular repeat cluster. According to BLAST analysis, *H. alpinum*, *H. theinum*, and *H. flavescens* had four common repeats (HF 5, HF 21, HF 145, and HF 186) that showed a 93–100% range in homology. Moreover, in *H. alpinum* and *H. flavescens*, two common repeats (HF 61 and HF 265) showed a 65–73% range in homology. At the same time, the relevant homologous repeats were not detected in *H. theinum*. In the repeatome of *H. alpinum*, two tandem repeats (HA 42 and HA 80), which were homologous with repeat HF 5, and two tandem repeats (HA 186 and HA 200), which were homologous with repeat HF 61, were detected.

Among the tandem DNA repeats identified in the genomes of *H. flavescens*, *H. alpinum*, and *H. theinum*, the homologous satDNAs HF 5, HA 42, HA 80, and HT 55 (29 bp) had the highest percent of genome proportion (0.29–1.2%). These repeats showed an 83–100% range in homology with the DNA repeats of *Medicago truncatula* (Fabaceae). One DNA repeat of *H. flavescens* (HF 21) and its homologous repeats revealed in the genomes of *H. alpinum* (HA 46) and *H. theinum* (HT 67) had the second largest genome proportion (0.35–0.69%) among the repeats identified in the genomes of the studied species (Table 1).

According to BLAST, HF 21, HA 46, and HT 67 showed a 73–89% range in homology with the DNA repeats of *Trifolium fragiferum* (Fabaceae). The DNA repeats HF 35 (53 bp) and HF 252 (217 bp) identified in the *H. flavescens* genome did not show homology with the repeats identified in the genomes of other studied species (detailed in Table 1). The genome proportions of repeats HF 145, HF 186, HF 265, and HF 252, as well as the relevant homologous repeats in *H. alpinum* and *H. theinum*, were considerably less, and according to BLAST, any repeat DNA sequences homologous to these repeats have not been identified in the available databases.

### 2.3. Karyotype Structure and Chromosomal Localization of 35S rDNA, 5S rDNA, and Satellite DNAs in the Studied Hedysarum Species

#### 2.3.1. FISH-Based Localization of 35S rDNA and 5S rDNA

**Figure 3:**
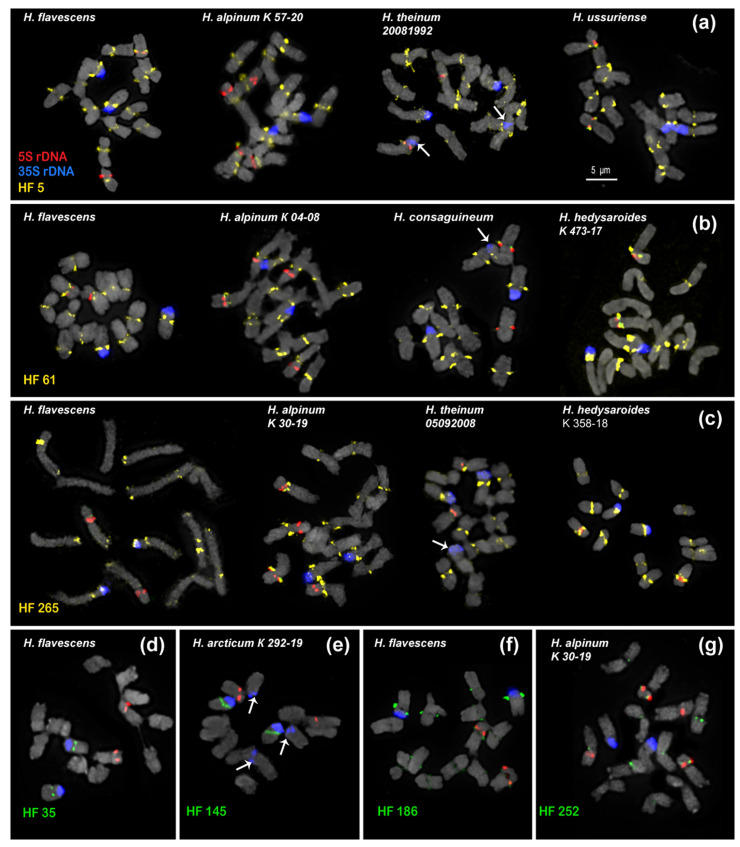
FISH-based localization of 5S rDNA, 35S rDNA, and satellite repeats on the chromosome spreads of the studied *Hedysarum* species. Metaphase spreads after FISH with HF 5 (**a**), HF 61 (**b**), HF 265 (**c**), HF 35 (**d**), HF 145 (**e**), HF 186 (**f**), and HF 252 (**g**). The correspondent probes and their pseudocolors are specified next to the metaphase spreads: *3*5S rDNA (blue); 5S rDNA (red); HF 5, HF 61, and HF 265 (yellow); HF 35, HF 145, HF 186, and HF 252 (green). Bar 5 μm.

**Figure 4:**
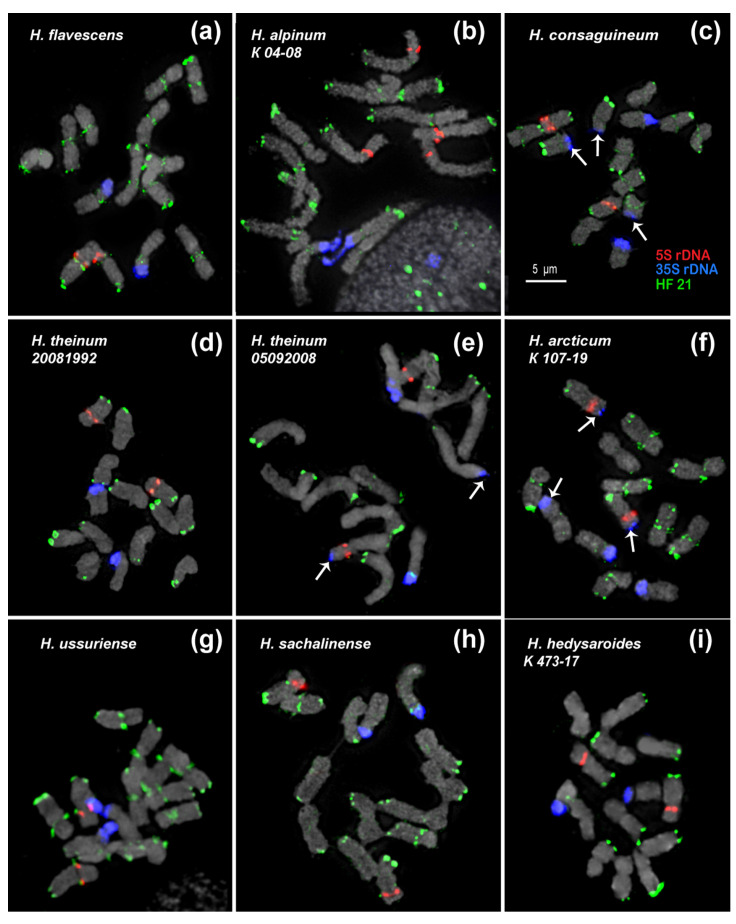
FISH-based localization of 5S rDNA (red), 35S rDNA (blue), and satellite repeat HF 21 (green) in the metaphase spreads of the studied accessions of *H. flavescens* (**a**), *H. alpinum* (**b**), *H. consaguineum* (**c**), *H. theinum* (**d**,**e**), *H. arcticum* (**f**), *H. ussuriense* (**g**), *H. sachalinense* (**h**), and *H. hedysaroides* (**i**). The correspondent probes and their pseudocolors are specified on the right. Arrows point to the polymorphic sites of 35S rDNA. Bar: 5 μm.

In the present study, we analyzed the chromosomal organization in the karyotypes of eight species of sect. *Hedysarum* using FISH with probes of 35S rDNA, 5S rDNA, and the identified satellite repeats (Figure 3 and Figure 4). It was revealed that the karyotypes of all studied species have 2n = 14 chromosomes, which are generally similar in chromosome morphology and localization of major 35S and 5S rDNA loci. The chromosome lengths were within the range of 1 to 7 µm. We found that the karyotypes of the diploid species, *H. sachalinense* and *H. ussuriense*, contained 2n = 14 chromosomes rather than 2n = 16 as previously reported [35,36] (Figure 4g,h).

**Figure 5:**
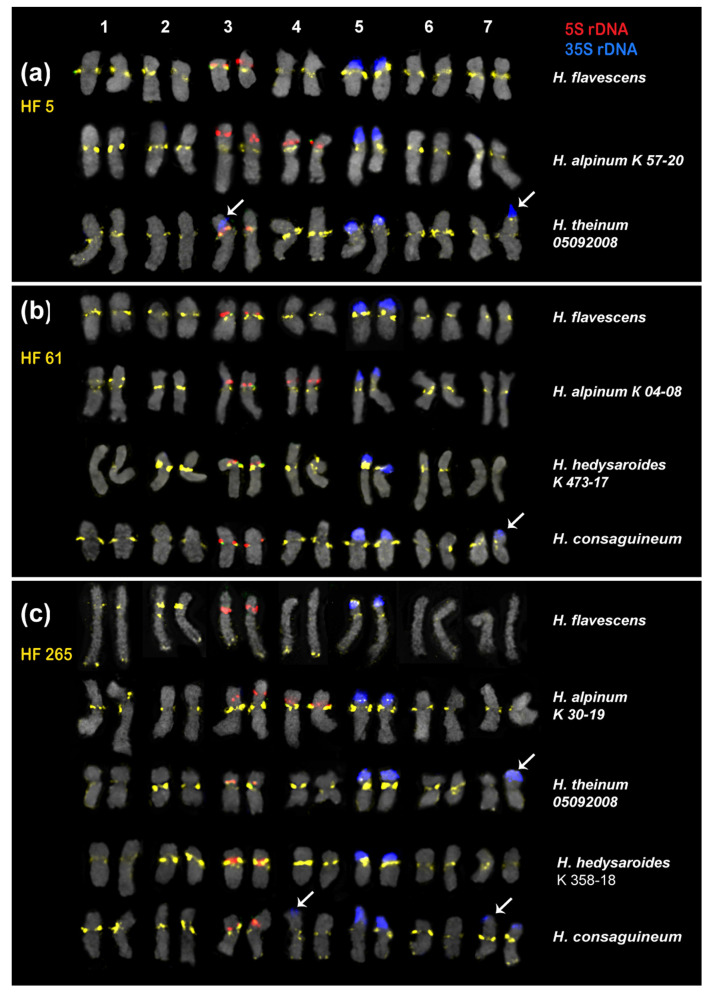
Karyotypes of the studied *Hedysarum* species after FISH with 5S rDNA, 35S rDNA, and satellite repeats (the same metaphase plates as in Figure 3): HF 5 (**a**), HF 61 (**b**), and HF 265 (**c**). Arrows point to the polymorphic sites of 35S rDNA. The correspondent probes and their pseudocolors are specified on the left: HF 5, HF 61, and HF 265 (yellow), and on the right: *3*5S rDNA (blue) and 5S rDNA (red).

**Figure 6:**
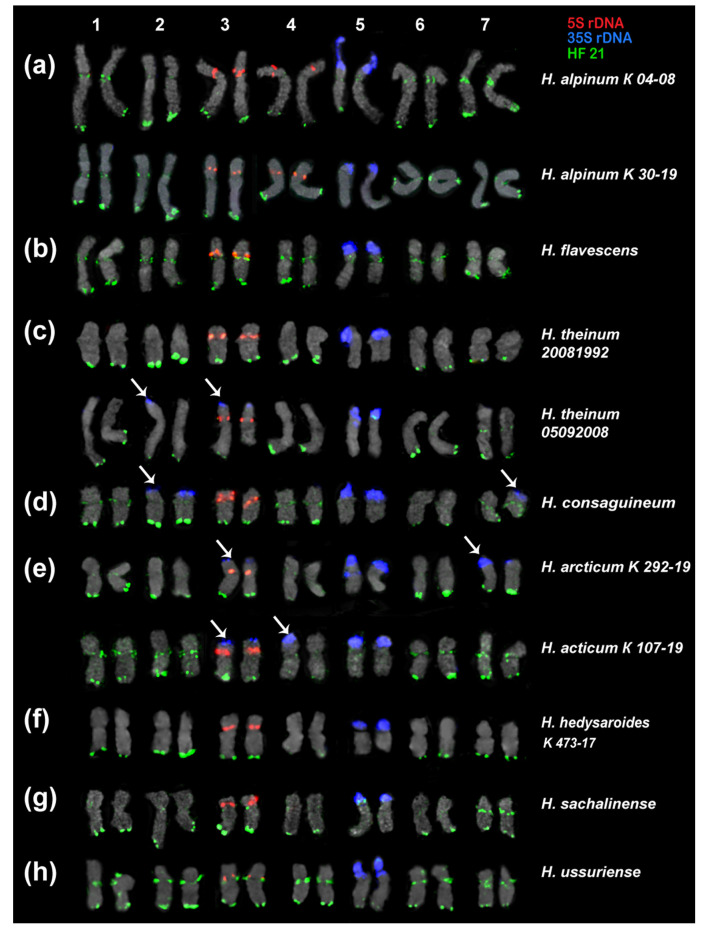
Karyotypes of the studied accessions of *H. alpinum* (**a**), *H. flavescens* (**b**), *H. theinum* (**c**), *H. consaguineum* (**d**), *H. arcticum* (**e**), *H. hedysaroides* (**f**), *H. sachalinense* (**g**), and *H. ussuriense* (**h**) after FISH with 5S rDNA (red), 35S rDNA (blue), and satellite repeat HF 21 (green) (the same metaphase plates as in Figure 4). Arrows point to the polymorphic sites of 35S rDNA. The correspondent probes and their pseudocolors are specified on the right.

Based on the chromosome morphology and patterns of chromosomal distribution of 35S rDNA, 5S rDNA, and the tandem DNA repeats, the karyograms of these species were constructed (Figure 5 and Figure 6). The karyotypes of the studied species contained one satellite (SAT) chromosome pair (5) bearing 35S rDNA sites and a pair of chromosomes with 5S rDNA sites localized in proximal regions of the short arms of chromosome pair 3. In *H. alpinum*, an additional 5S rDNA locus was detected on chromosome pair 4 (Figure 3g and Figure 4b). In *H. consaguineum* (Figure 4c), *H. arcticum* K 292-19 (Figure 3e), and *H. theinum* 05092008 (Figure 4e), different numbers of the additional minor 35S rDNA loci (polymorphic loci) were detected in the distal regions of chromosome pairs 2 and 7. In some karyotypes, a minor 35S rDNA locus was detected only on one homolog of chromosome pairs 2 and 7. In addition, in the karyotypes of some plants of *H. consaguineum* (Figure 5c) and *H. arcticum* K 107-19 (Figure 4f), the minor 35S rDNA locus was revealed in the distal region of chromosome pair 4. In one specimen, *H. theinum* 05092008 from the Altai Mountains (Figure 6c), and in all studied specimens of *H. arcticum* (Figure 6e), minor polymorphic loci of 35S rDNA were revealed in the distal regions of the short arms of chromosome pair 3.

#### 2.3.2. FISH-Based Localization of Satellite DNAs

For karyotype analyses of the species from sect. *Hedysarum*, we carried out FISH assays with oligonucleotide probes HF 5, HF 21, HF 35, HF 61, HF 145, HF 186, HF 252, and HF 265, which were designed based on eight DNA repeats identified in the *H. flavescens* genome (Figure 3 and Figure 4; Table 1).

According to the FISH results, two of the most common tandem repeats, HF 5 and HF 61, were localized in the pericentromeric regions of the species’ chromosomes (Figure 3a,b). The pericentromeric localization of these repeats made it possible to clarify the morphology of metacentric and submetacentric chromosomes in the karyotypes (Figure 5a,b).

Bright HF 5 clusters were observed on the chromosomes of all studied species. In *H. theinum*, HF 5 was also detected in the region of the large terminal DAPI band localized in the short arms of SAT chromosome pair 5 (Figure 5a). In *H. hedysaroides* and *H. theinum*, pericentromeric HF 61 signals were not detected (or were very weak) on chromosome pairs 1 and 7, although bright signals were observed in the karyotypes of other studied species (Figure 5b). HF 265 (the longest pericentromeric tandem DNA) presented large pericentromeric clusters on all chromosomes in the studied species (Figure 3c and Figure 5c). Moreover, additional polymorphic loci were observed in the terminal regions of several chromosomes in *H. flavescens* and *H. consaguineum* (Figure 5c). In the karyotypes of *H. hedysaroides* and *H. theinum*, HF 265 presented a very weak signal on chromosome pair 1 (similar to HF 61). In *H. flavescens*, *H. alpinum*, and *H. theinum*, an HF 265 cluster was detected in the distal region of SAT chromosome pair 5 (Figure 5c).

Short tandem repeats, HF 35 (53 bp) and HF 145 (51 bp), were localized only in the pericentromeric region of satellite chromosome 5 in all studied species (Figure 3d,e).

The DNA repeats with the smallest proportion in the genome, HF 186 and HF 252, presented very weak FISH signals in the pericentromeric regions of the chromosomes in the karyotypes of the studied species (Figure 3f,g).

HF 21 (179 bp) and its homologues HA 46 (176 bp) and HT 67 (188 bp) had a rather high proportion in the genomes of *H. flavescens*, *H. alpinum*, and *H. theinum*. We found that HF 21 could be the most promising marker for the identification of chromosomes and the assessment of chromosomal variability in *Hedysarum* species (Figure 4 and Figure 6).

In *H. flavescens*, HF 21 clusters were detected in the subtelomeric (except for chromosome pairs 5 and 6) and pericentromeric chromosome regions (Figure 4a). The most intense signals were observed on chromosome pairs 1–4 (Figure 6b).

In *H. alpinum* (Figure 4b and Figure 6a) and *H. ussuriense* (Figure 4g and Figure 6h), unlike the other species, bright hybridization signals were observed in the pericentromeric regions and the subtelomeric regions of the long arms of all chromosomes. The most intense subtelomeric signals were detected on chromosome 2.

In *H. consaguineum* (Figure 4c and Figure 6d) and *H. theinum* (Figure 4d,e and Figure 6c), bright hybridization signals were revealed in the subtelomeric regions of the long arms of chromosome pairs 1–4. The subtelomeric signal localized on chromosome pair 2 was the most intense. No subtelomeric signals were detected on SAT chromosome pair 5. On chromosome pairs 6 and 7, weak polymorphic signals were revealed in the subtelomeric chromosome regions.

In *H. consaguineum*, bright hybridization signals were observed in the pericentromeric chromosome regions of chromosome pairs 1 and 4, and polymorphic loci were detected on chromosome pairs 2, 3, 6, and 7 (Figure 6d).

In *H. theinum*, no pericentromeric hybridization signals of HF 21 were observed, and polymorphic loci of HF 21 were detected in the secondary constriction region of SAT chromosome 5 (Figure 6c).

In *H. hedysaroides* (Figure 6f) and *H. arcticum* (Figure 6e), bright hybridization signals of HF 21 were revealed in the subtelomeric regions of the long arms of chromosome pairs 1–3 (the brightest signals), 6, and 7.

In *H. arcticum* 107, polymorphic pericentromeric loci were detected on these chromosomes. In the karyotypes of *H. hedysaroides*, no pericentromeric hybridization signals of HF 21 were detected (Figure 6f).

In *H. sachalinense*, bright hybridization signals of HF 21 were revealed in the subtelomeric regions of the long arms on all chromosomes, except for SAT chromosome pair 5 (Figure 6g). Weak pericentromeric hybridization signals were observed on chromosome pairs 1–4 and 6. On chromosome pair 7, bright hybridization signals were detected on SAT chromosome pair 5, and a polymorphic locus was detected in the secondary constriction region.

**Figure 7:**
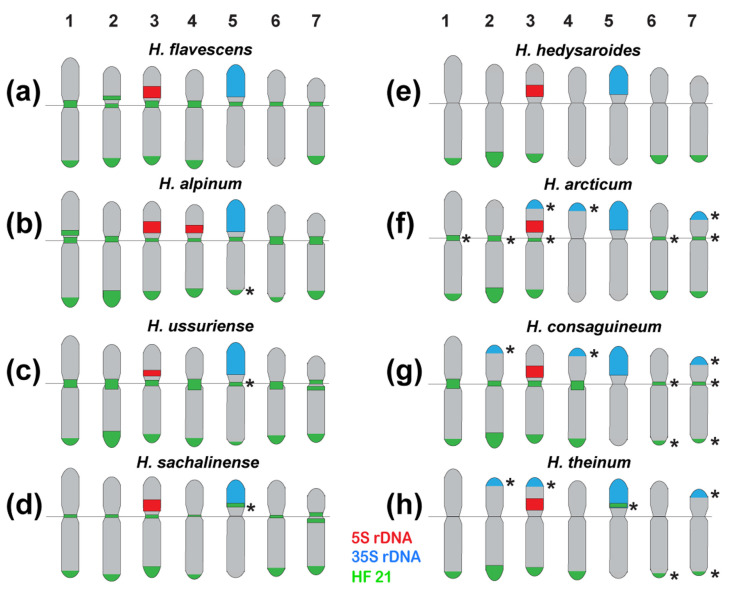
Species-specific idiograms of *Hedysarum* chromosomes showing the chromosomal distribution of the examined markers: 35S rDNA (blue), 5S rDNA (red), and HF 21 (green). *H. flavescens* (**a**), *H. alpinum* (**b**), *H. ussuriense* (**c**), *H. sachalinense* (**d**), *H. hedysaroides* (**e**), *H. arcticum* (**f**), *H. consaguineum* (**g**), and *H. theinum* (**h**). Asterisks indicate polymorphic sites.

For all studied species, the species-specific idiograms demonstrating the chromosomal distribution of HF 21, 35S rDNA, and 5S rDNA were constructed (Figure 7).

## 3. Discussion

Differential accumulation and loss of repetitive DNA are key drivers of genome size variation in flowering plants. Although satellite repeats can comprise up to 10–20% of the genome in some species, the bulk of the repeats typically consists of transposable elements [37,38]. In the present study, the comparative bioinformatic analysis of repeatomes of the species from sect. *Hedysarum* demonstrated high similarity in the repeatome composition of *H. alpinum*, *H. flavescens*, and *H. theinum*. Although the proportions of major repetitive DNA elements in the repeatomes of the studied species did not differ much, the lowest content of the repetitive DNA was found in the *H. theinum* genome (55.23%). In the studied species from sect. *Hedysarum*, LTR retrotransposons were the most common transposable elements. At the same time, the number of Ty3-gypsy elements was 3–4.5 times higher (depending on the species) than the number of Ty1-copia retroelements. As reported previously, in the species genomes from sect. *Multicaulia*, the number of Ty3-gypsy retroelements was 1.5–2 times higher than the Ty1-copia elements [18]. Between the genomes of the species from the sections *Hedysarum* and *Multicaulia*, the differences in the genome proportions of the Ty3-Gypsy superfamily were also detected. In the species genomes from sect. *Hedysarum*, two times more Ty3-Gypsy elements were detected compared to those from sect. *Multicaulia*. In related species, different types of repetitive DNA contribute to genome size evolution in a phylogenetic context. It was shown that 85% of the variations in genome size, which occurred between species in different lineages during the evolution within the tribe Fabeae, could be related to the differential accumulation of repetitive DNA, and the majority (57%) of these variations was due to one of the lineages of LTR retrotransposons, Ty3/gypsy [39].

Among satellite DNA families, a high rate of genomic changes was revealed, and they can be common to a group of related species or be species-specific [40]. Most species of the egume family are characterized by high variability in the number and diversity of satellite repeats [41,42,43]. In the species genomes from sect. *Hedysarum*, the genome proportion of satellite DNA was 1.21–2.41%, which was half that of the species from sect. *Multicaulia* (2.68–5.09%) [18]. In the Fabeae species genomes, satellite DNAs were shown to be the most evolutionarily dynamic repeats, which resulted in the emergence of highly divergent sequence families, and they could be present in one or a few closely related species and absent in others [41]. The common repeats were identified in the genomes of the studied species from sect. *Hedysarum*. They were localized in the pericentromeric chromosome regions and had no homology to any of the pericentromeric satDNA sequences of the genomes of the species from sect. *Multicaulia*. This is consistent with the high rates of changes (evolution) observed for satDNAs in different taxa [28,44] and indicates a distant relationship between the genomes of the species from the sections *Hedysarum* and *Multicaulia*. However, according to the performed comparative analyses, within sect. *Hedysarum* as well as within sect. *Multicaulia*, similarities in satellite DNA repeats were revealed. The genomes of *H. alpinum*, *H. flavescens*, and *H. theinum* contained four common repeats with almost the same length and 93–100% homology. In addition, in the genomes of *H. alpinum* and *H. flavescens*, two common repeats (HF 61 and HF 265) showed a high level of identity, which indicates the close relationship between the genomes of these species. This is confirmed by the similar chromosomal structures of the karyotypes of these species revealed in the present study and by our previous results of rapid GISHassays performed in *H. flavescens*, *H. alpinum*, and *H. theinum* [45].

In this study, the karyotypes of all studied species from sect. *Hedysarum* were represented by seven pairs of metacentric and submetacentric chromosomes. For *H. sachalinense* and *H. ussuriense*, 2n = 16 were previously reported [35,36]. However, those studies were conducted using monochrome chromosome staining. This method does not allow for accurate identification of chromosome pairs in the karyotypes, and the revealed additional chromosomes could be satellites detached from the SAT chromosomes or B chromosomes, which are often observed in the karyotypes of *Hedysarum* species [18]. The basic number of chromosomes, x = 7, is characteristic of sect. *Hedysarum*, which distinguishes this section from the other sections of the genus with x = 8 [11,15,16]. These data are consistent with the results of phylogenetic analysis of nuclear (ITS) and plastid DNA sequences (trnL-trnF and matK), which revealed two main lineages in the genus *Hedysarum*: the *Hedysarum* clade and the *Sartoria* clade [12,13,14]. In addition, the species from sect. *Hedysarum* differ from those of the sections *Multicaulia* and *Strachey* by a pronounced morphological differentiation and the carpological and anatomical structure of the fruit [1,11,46]. The listed facts indicate genomic differences in the species that belong to the sections *Multicaulia*, *Stracheya*, and *Hedysarum*. It was also previously shown that, despite the coincidence in the number of the main 35S rDNA and 5S rDNA sites detected in the species from the sections *Multicaulia* and *Hedysarum*, their localization on chromosomes was different, which also confirms the distant relationship among their genomes [18].

Ribosomal DNA is known to be one of the most abundant gene families and an unstable genomic region [47]. The reasons for this instability are not fully understood; however, rDNA loci have been reported as the predominant sites of repeated recombination [48]. This recombination between loci can cause both intragenomic variations in rDNA copy number and the amplification of new arrays [49]. It was previously reported that the loci of presumably inactive ribosomal RNA genes exhibit significant variability [50,51,52]. rDNA arrays and neighboring regions are also frequent targets for insertions of transposable elements [53]. The emergence of new 35S rDNA loci could occur due to intragenomic transposon activity, which can cause the translocation of rDNA copies to new genomic sites [54,55,56]. Within sect. *Hedysarum*, we found not only the species having major 35S rDNA and 5S rDNA sites in their karyotypes but also those containing both major and minor polymorphic (variable in number) loci of these genes. *H. alpinum* was the only species with additional minor 5S rDNA loci in its karyotype. This species was widespread but morphologically well-distinct [1,2]. The variability in chromosomal localization of minor 35S rDNA loci was detected in *H. theinum*, *H. arcticum*, and *H. consaguineum*, which grow in extreme environmental conditions.

It was previously shown that rDNA variations could affect rDNA transcription, which in turn might affect the translation of protein-coding genes and cell physiology [54,55,57]. Moreover, significant karyotypic variability in the number of minor 35S rDNA loci was revealed in plant populations adapted to certain climatic and geographical zones [58,59,60]. We also detected variability in number of minor 35S rDNA sites in the genome of *H. arcticum*, which grows in the harsh Arctic zone beyond the Arctic Circle. Other species (*H. theinum* and *H. consanguineum*) with polymorphic minor 35S rDNA loci are well adapted to climate conditions of high mountains, and they were distributed in a wide range of altitudes in the mountains and intermountain basins of Southern Siberia (from 1400 to 2700 m above sea level). Moreover, in these species, adaptation to extreme growing conditions was confirmed (with increasing altitude above sea level), even at the level of anatomical features of their leaf blade structure [61]. Thus, our results are consistent with the earlier reported data on the influence of stressful environmental conditions on the occurrence of rDNA variability; in turn, rDNA variability could increase the ecological adaptability of some organisms [55].

For various plant species, localization of satDNAs in the pericentromeric and subtelomeric chromosome regions is a characteristic feature [25,62,63]. In the studied species from sect. *Hedysarum*, three satDNAs (HF 5, HF 61, and HF 265) were distributed in the pericentromeric chromosome regions of *Hedysarum*, which made it possible to clarify the morphology of their chromosomes. In the karyotypes of species from sect. *Multicaulia*, most satellite repeats were also localized in the pericentromeric chromosome regions [18]. At the same time, according to BLAST, the sequence homology between the pericentromeric satDNAs from both sections *Hedysarum* and *Multicaulia* was not revealed. In most species from the tribe Fabeae, species-specific pericentromeric satDNAs were found among the 64 pericentromeric satDNA superfamilies [41,64].

In all the studied species, two short tandem repeats, HF 35 and HF 145, were localized only in the pericentromeric regions of SAT chromosome pair 5. The homology with other pericentromeric repeats of the species from sect. *Hedysarum* was not detected. The chromosome-specific repeats were also identified in other plant species [65,66].

The evolutionary rate of tandem repeat satDNA was shown to be higher than in other genomic sequences. It is believed that the mechanisms leading to rapid turnover of these sequences promote genomic reorganization [26].SatDNAs are usually variable in genomic abundance and in patterns of their distribution on chromosomes of related species within the genus [25,27,67]. This could be the result of an increase or decrease in number of repeat copies in genomes, just as it was found in *Poa* species [68]. Within the tribe Fabeae, different variations in satDNAs were revealed in each of the identified satDNA superfamilies [41,64]. They are often associated with heterochromatin, which can be localized in different chromosome regions. FISH satDNA chromosome patterns make it possible to identify homologous chromosomes, recognize chromosomal rearrangements, and detect differences between lineages and species [26,27,28]. In the present study, a high level of homology of some satDNA sequences allowed us to use them as FISH oligonucleotide probes in comparative karyotype analyses of the related *Hedysarum* species. The chromosomal distribution patterns of 35S rDNA, 5S rDNA, and satDNA HF 21 were species-specific in the karyotypes of the studied species from sect. *Hedysarum*, and significant interspecific variability in satDNA hybridization signals was observed in the pericentromeric and subtelomeric regions of the chromosomes. Within sect. *Multicaulia*, to identify species-specific patterns in karyotypes and study interspecific diversity, molecular chromosomal markers 35S rDNA, 5S rDNA, and satDNA Hz 6 were previously developed [18].

Tandem repeats, rDNA and satDNA, are considered a fast-evolving fraction of the repeatome that can diverge in both copy number and sequence between closely related species [41,64]. All satDNAs have a variable length of the repeat unit (monomer) and form tandem arrays [26,28]. The sequences of satellite monomers evolve concertedly through a molecular drive. As a result, mutations are homogenized in a genome and become fixed in populations, which results in satDNA monomer homogeneity maintenance within a species during evolution [69]. Some satDNA sequences demonstrate sequence conservation for long evolutionary periods [40]. Since many satellite DNAs exist in a genome, the evolution of species-specific satDNAs might result from copy number changes within a library of satellite sequences common for a group of species [40,69]. Among species from sect. *Hedysarum*, the revealed differences in the chromosomal distribution of satDNA HF 21 could also be related to the speciation process.

Some authors believed that *H. arcticum* was a subspecies of *H. hedysaroides* (*H. hedysaroides* (L.) Schinz et Thell. ssp. *arcticum*) (B. Fedtsch.) P.W. Ball [2,20]. In other studies, *H. arcticum* was considered an independent species due to its morphological and geographical isolation [1,19]. Phylogenetic analyses of the nrDNA ITS and plastid data showed that *H. hedysaroides* and *H. arcticum* were included in one clade, which indicated their close relationships [70]. According to our results, the patterns of chromosomal distribution of 35S rDNA and HF 21 in *H. arcticum* and *H. hedysaroides* were different. In addition, in the studied specimens of *H. arcticum*, high variability in number of minor 35S rDNA loci was revealed, which is consistent with the assumption on the specific isolation of *H. arcticum.*

*H. sachalinense*, distributed on Sakhalin Island, is considered to be an independent species [32]. At the same time, some taxonomists described *H. sachalinense* as a subspecies of *H. hedysaroides* (*H. hedysaroides* subsp. *sachalinense* (B. Fedtsch.) Vorosch. [71]. In this study, a karyotype of *H. sachalinense* differed from *H. hedysaroides* by the presence of HF 21 clusters in the pericentromeric regions of the chromosomes. These differences are consistent with the taxonomist’s assumption that *H. sachalinense* and *H. hedysaroides* are closely related but separate species [32].

The alpine species *H. theinum* and *H. flavescens* have overlapping habitats in the Tian-Shan and Pamir-Alay Mountains [19,31]. However, these species have individual distribution patterns of 35S rDNA and HF 21 clusters in their karyotypes. Our results are consistent with the previous phylogenetic studies that placed *H. theinum* and *H. flavescens* in one clade but included in different subgroups [12,70].

In the karyotypes of both *H. alpinum* and *H. ussuriense*, we observed clusters of HF 21 in the pericentromeric and subtelomeric regions of chromosomes, which were not revealed in other studied species. According to the previous phylogenetic data, the plastid tree strongly supported separate groups, which included *H. alpinum* and *H. ussuriense* [12,70]. This is generally consistent with our results since we detected unique minor 5S rDNA loci in the karyotype of *H. alpinum*.

Thus, satDNA HF 21, together with 35S rDNA and 5S rDNA, could be used as valuable molecular chromosomal markers to analyze intra- and interspecies chromosomal variability in karyotypes and clarify the taxonomy of the sect. *Hedysarum* species and the relationships among their genomes.

## 4. Materials and Methods

### 4.1. Plant Materials

In the present study, we examined thirteen plant accessions covering eight species of the sect. *Hedysarum* obtained from different seed sources (detailed in Table 2), including the germplasm collections of the All-Russian Institute of Medicinal and Aromatic Plants (AIMAP), Moscow, Russia, and the Central Siberian Botanical Garden (CSBG), SB RAS, Novosibirsk, Russia. Wild *Hedysarum* accessions were collected and identified by Dr. I. Yu. Selyutina and S.V. Volodarskaya (CSBG), Dr. V.V. Shejko (Botanical Garden-Institute FEB RAS, Vladivostok, Russia), and S.Y. Dr. Syeva (Gorno-Altay Research Institute of Agriculture, Altay Republic, Russia) (Table 2). The morphological description of eight studied *Hedysarum* species is presented in Table S3.

### 4.2. Sequence Analysis and Identification of DNA Repeats

The genomic DNAs of *H. alpinum*, *H. flavescens*, and *H. theinum* were isolated from young leaves using the CTAB method with minor modifications [72]. Genome DNA low-coverage sequencing was performed at the Beijing Genomics Institute (BGISeq platform) (Shenzhen, China) according to the NGS protocol for generating 5–10 million paired-end reads of 150 bp in length, which was at least 0.5–0.9× of the coverage of the *Hedysarum* genome (1C = 1643 Mbp) [17]. The raw data were uploaded to the NCBI database (http://www.ncbi.nlm.nih.gov/bioproject/1037005, accessed on 8 November 2023). The comparative integrated bioinformatic analysis of the repeatomes of *H. alpinum*, *H. flavescens*, and *H. theinum* was performed using RepeatExplorer/TAREAN pipelines [34]. For each studied species, the genomic reads were filtered by quality. Then 1,000,000 high-quality reads were randomly selected for further analyses, which corresponds to 0.09× of the coverage of the genome *Hedysarum* [17] and is within the limits recommended by the developers of these programs (a genome coverage of 0.01–0.50× is recommended) [34]. RepeatExplorer/TAREAN was launched with the preset settings based on the Galaxy platform (https://repeatexplorer-elixir.cerit-sc.cz/galaxy/, accessed on 8 November 2023). The default threshold is explicitly set to 90% sequence similarity spanning at least 55% of the read length (in the case of reads differing in length, it applies to the longer one). The sequence homology of the identified tandem DNA repeats was estimated by BLAST (NCBI, Bethesda, MD, USA). Eight abundant tandem DNA repeats of *H. flavescens*, which exhibited high sequence homology with seven DNA repeats of *H. alpinum* and four DNA repeats of *H. theinum*, were used for generating oligonucleotide FISH probes (see Supplementary Table S2) with Primer3-Plus software [73].

### 4.3. Chromosome Spread Preparation

The seeds of the studied *Hedysarum* species were scarified, then kept in hot (85–90 °C) water for 5–10 min, then germinated in Petri dishes for a week at 22 °C. Excised root tips (0.5–1 cm long) were incubated in ice water for 16–24 h for accumulation of mitotic cells. The root tips were fixed in ethanol:acetic acid (3:1) for 3–24 h at room temperature and then placed in a 1% acetocarmine solution for 15 min for good chromosome spreading (in 45% acetic acid). One root tip was cut on the slide, macerated with a dissecting needle in a drop of 45% acetic acid, and covered with a cover slip. After squashing and freezing in liquid nitrogen, the slides were dehydrated in 96% ethanol and air-dried.

### 4.4. FISH Procedure

In the FISH assays, we used two wheat DNA probes: pTa71 containing 18S-5.8S-26S (35S) ribosomal DNA (rDNA) and pTa794 containing 5S rDNA [74,75]. These DNA probes were labeled directly with fluorochromes Aqua 431 dUTP or Red 580 dUTP (ENZO Life Sciences, New York, NY, USA) using the Nick Translation DNA Labeling System 2.0 (Life Sciences Inc., New York, NY, USA). Additionally, we used oligonucleotide probes HF 5, HF 21, HF 35, HF 61, HF 145, HF 186, HF 252, and HF 265, which were synthesized with the labeled nucleotides Cy3-dUTP or 6-FAM-dUTP in Syntol (Moscow, Russia). Before the fiFISH procedure, chromosome slides were pretreated with 1 mg/mL of RNase A (Roche Diagnostics, Mannheim, Germany) in 2 × SSC at 37 °C for 1 h. Then the slides were washed three times for 10 min in 2 × SSC, dehydrated in the graded ethanol series (70%, 85%, and 96%), and air-dried. The hybridization mixture (50% formamide, 70% of hybridization specificity (stringency)) containing 40 ng of each labeled probe was added to each slide (22 μL). Coverslips were placed on the slides and sealed with rubber cement. Slides with DNA probes were co-denatured at 74 °C for 4 min, placed in a moisture chamber, and hybridized overnight at 37 °C. After the FISH procedure, the slides were placed in 0.1 × SSC at 50 °C for 5 min, 2 × SSC at 37 °C for 10 min, 2 × SSC at RT for 5 min, and 1 × PBS at RT for 5 min. Then, the slides were dehydrated in the graded ethanol series, air-dried, and stained with 0.1 μg/mL DAPI (4′,6-diamidino-2-phenylindole) (Serva, Heidelberg, Germany) in Vectashield mounting medium (Vector Laboratories, Peterborough, UK).

### 4.5. Chromosome Analysis

At least five plants of each accession and fifteen metaphase plates from each specimen were examined. The chromosome slides were analyzed with the Olympus BX 61 epifluorescence microscope with a standard narrow band pass filter set (Olympus, Tokyo, Japan). Images were captured with a monochrome charge-coupled device camera (Cool Snap, Roper Scientific, Inc., Sarasota, FL, USA). Then they were processed using Adobe Photoshop 10.0 software (Adobe, Birmingham, AB, USA).

## 5. Conclusions

In the present study, the comparison of repeatomes of *H. alpinum*, *H. theinum*, and *H. flavescens* using NGS data, bioinformatic technologies, and FISH chromosome mapping of 35S rDNA, 5S rDNA, and the identified satDNAs allowed us to provide new insight into the karyotype structures and genomic diversity within sect. *Hedysarum* and clarified the systematic position of *H. sachalinense* and *H. arcticum*. Our results confirmed the close genomic relationships of species within sect. *Hedysarum* and distant relationships of the species belonging to the different sections *Hedysarum* and *Multicaulia*. Our findings are important for further comparative genome studies within the genus *Hedysarum*.

## Figures and Tables

**Table 1:** Homology of the tandem repeats ^1^ identified in the genomes of *H. alpinum*, *H. flavescens*, and *H. theinum*, and the FISH-based patterns of their chromosomal distribution.

Tandem Repeat/Genome Proportion, %			Repeat Length, bp	Blast Homology	Chromosome Localization
H. flavescens	H. alpinum	H. theinum			
**HF 5/1.2**	HA 42/0.49 (93% identity with HF 5) HA 80/0.29	HT 55/0.39 (95% identity with HF 5)	29 (HF 5, HA 42, HT 55) 59 (HA 80)	83–100% identity with *Medicago truncatula*, chromosome: 5 clone mte1-70c15, Sequence ID: CT009651.1	Pericentromeric regions
**HF 21/0.69**	HA 46/0.45 (94% identity with HF 21)	HT 67/0.35 (97.93% identity with HF 21)	179 (HF 21) 176 (HA 46) 188 (HT 67)	73–89% identity with *Trifolium fragiferum* genome assembly, chromosome: 1 Sequence ID: X940789.1	Subtelomeric and pericentromeric regions of some chromosomes
**HF 35/0.51**	no	no	53	no	Pericentromeric regions of satellite chromosomes
**HF 61/0.32**	HA 186/0.014 (84% identity with HF 61) HA 200/0.012 (73% identity with HF 61)	no	167 (HF 61) 189 (HA 186) 189 (HA 200)	no	Pericentromeric regions
**HF 145/0.074**	HA 211/0.011 (97% identity with HF 145)	HT 218/0.027 (97% identity with HF 145)	51	no	Pericentromeric regions of satellite chromosomes
**HF 186/0.029**	HA 154/0.019 (97% identity with HF 186)	HT 234/0.019 (96.12% identity with HF 186)	168 (HF 186) 200 (HA 154) 178 (HT 234)	no	Weak signals in the pericentromeric regions of some chromosomes
**HF 265/0.013**	HA 171/0.028 (65% identity with HF 265)	no	651 (HF 265) 144 (HA 171)	no	Pericentromeric regions; subtelomeric and pericentromeric regions of some chromosomes: *H. flavescens* and *H. consaguineum*
**HF 252/0.014**	no	no	217	no	Weak signals in the pericentromeric regions of some chromosomes

^1^ Each tandem repeat was designated as follows: HF ### for *H. flavescens*, HA ### for *H. alpinum*, and HT ### for *H. theinum*, where ### is the numerical name of a particular repeat cluster. The names of the repeats used as FISH probes are specified in bold type.

**Table 2:** List of the studied *Hedysarum* accessions.

Species	Voucher/Origin
*H. alpinum* L.	K 57-20/Russia, Kirov, Botanical Garden of Vyatka State University/germplasm collection of AIMAP, 2020
*H. alpinum* L.	K 04-08/germplasm collection of AIMAP, 2023
*H. alpinum* L.	K 30-19/Russia, Tomsk, Botanical Garden of Tomsk State University/germplasm collection of AIMAP, 2023
*H. arcticum* B. Fedtsch.	K 107-19/Russia, Volgograd Regional Botanical Garden (VRBG)/germplasm collection of AIMAP, 2019
*H. arcticum* B. Fedtsch.	K 292-19/Russia, Volgograd Regional Botanical Garden (VRBG)/germplasm collection of AIMAP, 2019
*H. consanguineum* DC.	AKT25082004/N49.90941, E088.03343 Russia, Altai region, Kosh-Agachsky district, Beltir village, right bank of the Taldura River/Collected by Selyutina I. Yu. 25.08.2004
*H. flavescens* Rgl. et Schmalh.	CSBG_Fl_U30072007/Sweden, Uppsala, Botanical Garden of University of Uppsala/germplasm collection of CSBG, 2022
*H. hedysaroides* (L.) Schinz et Thell.	K 473-17/Austria, Botanical Garden of University of Vienna/germplasm collection of AIMAP, 2017
*H. hedysaroides* (L.) Schinz et Thell.	K 358-18/Austria, Botanical Garden of University of Vienna, germplasm collection of AIMAP, 2018
*H. sachalinense* B. Fedtsch.	SMZ05102021/N48.24823, E142.58493 Russia, Sakhalin region, Makarovsky district, khr. Zhdanko, rocky slope/Collected by Shejko V.V. 05.10.2021
*H. theinum* Krasnob.	CSBG_Fl_KVKRI(I)20081992/N50.31667, E 083.85194 East Kazakhstan, Ridder district, Ivanovsky Ridge, subalpine meadow/Collected by Volodarskaya S.V. 25.08.1992/germplasm collection of CSBG, 2008
*H. theinum* Krasnob.	CSBG_Fl_AUSK(I)05092008/N50.04391, E 085.12013 Russia, Altai region, Krasnaya Mountain, subalpine meadow/Collected by Syeva S.Y. 05.09.2008/germplasm collection of CSBG, 2010
*H. ussuriense* I. Schischk. et Kom.	K 145-21/France, Lautaret Alpine Garden/germplasm collection of AIMAP, 2021

## Data Availability

All data generated or analyzed during this study are contained within the article and Supplementary Materials.

## Supplementary Materials

The following supporting information can be downloaded at: https://www.mdpi.com/article/10.3390/ijms252212340/s1.
