# Supplementary material for: Repeatome Analysis and Satellite DNA Chromosome Patterns in Hedysarum Species
Source: Int J Mol Sci. 2024 Nov 17;25(22):12340. doi: 10.3390/ijms252212340 (PMC11595117; doi:10.3390/ijms252212340)
Supplement: Supplementary file 1 [file ijms-25-12340-s001.zip › ijms-3290031-supplementary.pdf]

Supplementary Table S1. Proportion of Major Repetitive DNA Sequences Identified in Genomes of the Studied *Hedysarum* Species.

Repeat Name	Genome proportion (%)		
	H. flavescens	*H. theinum*	*H. alpinum*
**Retrotransposons (Class I)**	**38.09**	**41.96**	**45.44**
**Ty1 Copia**	**5.8**	**9.69**	**10.99**
Ale	0.5	0.23	0.23
Angela	0.95	0.53	0.68
Bianca	0.01	-	-
Ivana	-	0.2	0.11
SIRE	3.96	8.26	9.51
TAR	0.38	0.37	0.39
Tork	-	0.1	0.07
**Ty3-Gypsy**	**26.84**	**32.1**	**33.84**
non-chromovirus Athila	3.71	11.61	9.24
non-chromovirus Tat- Ogre	1.89	1.74	2.17
non-chromovirus Tat-Retand	0.05	0.5	1.03
chromovirus CRM	0.05	0.3	0.36
chromovirus Tekay	20.69	17.95	21.04
**LINE**	**0.2**	**0.13**	**0.15**
**Unclassified LTR elements**	**5.25**	**0.04**	**0.46**
**Transposons (Class II)**	**5.45**	**5.8**	**7.44**
Cacta	5.33	5.26	6.91
MuDR_Mutator	0.12	0.53	0.51
hAT	-	0.01	0.01
PIF_Harbinger	-	-	0.01
**rDNA**	1.43	1.45	0.95
**Unclassified repeat**	6.61	4.96	5.37
**DNA satellite**	2.41	0.89	1.33
**Organelle**	5.3	6.87	2.93
**Putative satellites**	9 high confident<br>3 low confident	6 high confident<br>3 low confident	6 high confident<br>3 low confident

Supplementary Table S2. List of the oligonucleotide FISH probes.

Tandem Repeat	Oligo FISH probe name/ length, bp	Oligo FISH probe sequence
HF 5	HF 5/29	TAGTCACGATATGCTTCAATGTGTCAAAA
HF 21	HF 21/62	AACTTGCAAAATCCTAAAATTTGTAGTCTATAGCC TTTGAAAATTGAGAAAAAACCATAA
HF 35	HF 35/53	GGTTTGATAGTTTTCGAAGGCAAGTTTCAAATT TTGTCCTGAAT
HF 61	HF 61/64	GAA AATATGCCTTTTGACAGTTTTCTCACACAT TTTCGTATTTAACCACCGCACATGCACATGT
HF 145	HF 145/46	CTAAACAAGACTTGTTAGCACTACTTTAGGGTTCT TAGGCCTAGTC
HF 186	HF 186/52	GGTGAACATGGAAAGGCAGTGTCTTGGTGACATG TAGGTGCAAAGGGGTCAG
HF 265	HF 265/60	TGGATGAAGATCCAACGGTCGGATCTTGAGGCCG ATGTTTCTAATCTTCACCACTGAGTG
HF 252	HF 252/50	TAAACCTTGGTGAAGGTGGCAATCTATGGCTTTGT GATTTTATTAATATG

Supplementary Table S3. Morphological description of eight studied *Hedysarum* species.

Species	*H. alpinum* [1,19]	*H. arcticum* [1,19]	*H. hedysaroides* [2,20]	*H. consanguineum* [19]	*H. sachalinense* [32]	*H. flavescens* [3,31]	*H. ussuriense* [32]	*H. theinum* [19,30]
Plant height, cm	40-120(150)	10(15)-50(60)	35(50)	10-45	50-60	Up to 150	40-50	45-90
Number of pairs of leaflets	5-11	(3)4-9(10)	6-9	4-8	6-12	3-5	7-12	6-9(10)
Leaflets length, cm	1.5-3.5	1.2-3(4)	1.2-2	1-2.5(3.5)	1-2.5(3.5)	2(3.5)-4.5	0.7-1.5	2.5-5
Leaflets width, cm	0.5-1.2	0.4-1.2(1.5)	0.4-0.8	0.5-1.1(1.5)	0.5-1.1(1.5)	1.7-2(3.5)	0.3-0.4	0.5-1
Leaf shape	Oblong-lanceolate, elongate-elliptic, oblong-ovoid	Oblong-ovate-elliptic, elliptic-lanceolate	Ovate or oblong-ovate, less often lanceolate, blunt or rounded at the apex	Elliptic, oblong-elliptic	Pointed-elliptical or ovate-oblong	Oblong-ovate or elliptic, rounded or blunt at the apex with a point	Ovate	Oblong, oblong-lanceolate.
Inflorescence length, cm/<br><br>Number of flowers per	5-15(20)/<br><br>10-40	4-10, with fruits up to 16cm long/	3-7(12)/<br><br>15-30(40)	(3)4-9/<br><br>(10) 27-30	3-7/<br><br>25-35	15-35/<br><br>15-35	5-10/<br><br>6-20	4-7(13) cm long, with fruits up to 20 cm long/

raceme		5-30						20-40
Bracts length, mm/ Bracts shape	4-6 Linear-lanceolate, lanceolate 1.5-3 times shorter than pedicels, less often - almost equal	Lanceolate, linear-lanceolate.	Linear-lanceolate, 5-7 mm long, 0.5-1(1.5)mm wide, longer than the calyx tube, in lower flowers longer than the whole calyx.	lanceolate, reach or exceed the teeth of the calyx	5-7 lanceolate-awl-shaped, shorter calyx	3-5 Towards the end of flowering shorter pedicels	linear-filamentous	Narrowly lanceolate, almost linear, 4-10(12) mm long, bracts linear 2-5 mm long.
The calyx and its teeth length, mm	3.5-4.5 pubescent with short hairs, lower teeth slightly shorter than the calyx tube, upper teeth are 2-4 times shorter than it	5-7 teeth triangular-lanceolate, lanceolate, 1.5-3(4) times shorter than the tube	5-7 pubescent, denticles triangular-oblong, upper ones shorter than the tube, lower ones almost equal in length to the tube.	3.5-4.5 triangular or triangular-lanceolate, 1.5-2.5 times or slightly shorter than the tube	7-9 The tube is pressed-greyish the teeth are lanceolate-triangular, 3-5 mm long, shorter than or equal to the tube	4-6 Bell-shaped, lower calyx teeth longer than the tube, upper teeth shorter than the tube	the calyx is bell-shaped, the teeth are triangular, everything is shorter than the tube	5-9 mm long, teeth filiform, filiform, with triangular base, short ones almost equal to the tube, the longest ones 1.5-2 times longer than it

Corolla colour	Pink, mauve-pink	Purple, violet-red.	Dark or light purple	Purple or bright pink	Crimson or purple	Yellow	Pale yellow	Lilac, pink and purple.
Corolla size, mm<br><br>Corolla dimensions	13-15(16)<br><br>boat longer than flag, wings almost equal to flag	16-25(27)<br><br>flag 1(2)-4(5) mm shorter than boat. Wings almost equal to or shorter than flag	16-18(21)<br><br>oblong, notched at apex, wings 16-18 mm long, peduncle 17-19(21)mm long	(5)16-19мм<br><br>the wings are equal to the flag or slightly shorter than it	16-18(20)<br><br>flag on the top is notched, equal to or shorter than the boat	15-20, flag oblong or oblong-elliptic, slightly marginate at apex, boat longer than the flag and wings.	the flag is shorter than the wings, the wings are shorter than the boats	14-20mm long. Boat slightly longer than the flag. Wings almost equal to or slightly shorter than boat
Loments	1-4 segments<br><br>oval-elliptic or oval, reticulate with very narrow border, less often without it, glabrous or scatteredly pubescent	2-6 segments, roundish-elliptic or oblong, thin-setose, broadly bordered along the margin, glabrous, sometimes with sparse hairs along one suture	2-4 segments, oblong-oval, glabrous, thinly setose, margin with comparatively broad wing	3-6 segments , thinly setose, margin with broad wing	4-6 segments, thinly setose, margin with broad wing	2-4 flat segments, oblong-elliptic or oblong, thinly setose, margins with broad, entire wing, narrowed towards base.	2-3 segments,<br><br>margin with narrow wing	In number 3-5, pubescent, margins with well-defined border
